# Treatment of environmental contamination using sepiolite: current approaches and future potential

**DOI:** 10.1007/s10653-020-00705-0

**Published:** 2020-09-11

**Authors:** Na Song, Andrew Hursthouse, Iain McLellan, Zhenghua Wang

**Affiliations:** 1 School of Computing, Engineering and Physical Sciences, University of the West of Scotland, Paisley, PA1 2BE UK; 2 Hunan Provincial Key Laboratory of Shale Gas Resource Utilization, Hunan University of Science and Technology, Xiangtan, 411201 China

**Keywords:** Modified sepiolite, Adsorption, Potentially toxic elements, Remediation material composition, Economic analysis

## Abstract

To evaluate the potential of sepiolite-based materials to resolve environmental pollution problems, a study is needed which looks at the whole life cycle of material application, including the residual value of material classified as waste from the exploitation of sepiolite deposits in the region or from its processing and purification. This would also maximize value from the exploitation process and provide new potential for local waste management. We review the geographical distribution of sepiolite, its application in the treatment of potentially toxic elements in soil and across the wider landscape, an assessment of modification and compositional variation of sepiolite-based applications within site remediation and wastewater treatment. The potential of sepiolite-based technologies is widespread and a number of processes utilize sepiolite-derived materials. Along with its intrinsic characteristics, both the long-term durability and the cost-effectiveness of the application need to be considered, making it possible to design ready-to-use products with good market acceptance. From a critical analysis of the literature, the most frequently associated terms associated with sepiolite powder are the use of lime and bentonite, while fly ash ranked in the top ten of the most frequently used material with sepiolite. These add improved performance for the inclusion as a soil or wastewater treatment options, alone or applied in combination with other treatment methods. This approach needs an integrated assessment to establish economic viability and environmental performance. Applications are not commonly evaluated from a cost–benefit perspective, in particular in relation to case studies within geographical regions hosting primary sepiolite deposits and wastes that have the potential for beneficial reuse.

## Introduction

Environmental contamination is a persistent issue bringing more challenges to the health and development of the world (Uddin 2017). Pollution caused by the excessive presence of potentially toxic elements (PTEs) is one of the common environmental issues and relates to soil, water, sediment, and air. The magnitude of the total release is difficult to accurately estimate given the diverse point and diffuse sources associated with human activity. However, it is reported that an estimated 8.2% of investigated arable land (7.59 million ha) in China is contaminated with PTEs due to (i) high natural concentrations (local geology), (ii) secondary enrichment in soil-forming process and (iii) human activities (China Geological Survey 2015). The environmental problems caused by PTEs are not only affected by their concentrations but also by the different chemical species present under varying environmental conditions. The persistence of PTEs in the environment is prolonged because of resuspension and circulation in the atmosphere and transfer between terrestrial and aquatic systems (Shotyk et al. 2003). This is particularly true in areas of mining, ore exploitation, and metallurgical activity where in addition to direct soil deposition, rivers and wetlands have been contaminated by the PTEs. In addition to the PTEs content, hazards from PTEs are also affected by environmental conditions. In areas where soil PTEs exceed regulatory standards due to high geological background without human activity, the impact from soil PTEs is relatively low and transfer in the food chain is not significant. It is easier for crops to absorb and accumulate PTEs in soils that have been introduced through human activity, making these sources, in general, more accessible to wider dispersal due to chemical reactivity and bioavailability (China Geological Survey 2015). With the changes of soil environmental conditions, a high pollution level of soils with PTEs may lead to migration of contaminants into adjacent areas, which consequently generate hazard to the environment and living organisms (Twaróg et al. 2020). High quantities of PTEs existed in the wastewater from mining, chemical processing plants. The contamination was discharged into the environment as stable forms. Where PTE ions are stable in wastewater or other contaminated aqueous environments, they can be ingested and accumulated easily by aquatic and terrestrial organisms as they are not biodegradable, with the potential to threaten human health through transfer in the food chain (Ajao et al. 2020).

In the management of industrial wastewater, attention has been paid to the treatment of PTEs, such as Cu, Zn, Pb, Cd, Hg, As, Cr and Ni (Ihsanullah et al. 2016) as these are the PTEs more strongly influenced by human activity. Many physical, chemical and biological techniques have been applied to remove PTEs from wastewater including chemical precipitation, physical adsorption, electrolysis, biological accumulation, reverse osmosis and ion exchange (Ihsanullah et al. 2016). Adsorption is one of the most viable methods for pollution treatment, owning to its relatively straightforward application, high efficiency and insensitivity to the toxicities of contaminants. Activated carbon is probably one of the most widely applied adsorbents with many examples of application to remediate contaminated soil (Sörengård et al. 2019), sediment (Rakowska et al. 2014), atmosphere (Huang et al. 2019) and water (Hadi et al. 2015). More novel carbonaceous materials have also been studied for application in land/soil/sediment treatment, including a wide range of nanomaterials (Shahamirifard et al. 2016), such as graphene oxide (Isari et al. 2018; Zhao et al. 2011) and carbon nanotubes (Tofighy and Mohammadi 2015). However, these systems often require extensive synthesis, purification, and as viable treatment technologies still require further optimization. In pursuit of solutions within the life cycle approach to reduce the environmental impact of technologies, more inexpensive and low-cost materials still need to be sought.

Currently, clay minerals are a matter of much attention because they have been utilized as effective amendments for soil contaminated with PTEs and low-cost adsorbents for removing PTEs ions in aqueous solution (Uddin 2017; Xu et al. 2017). As a commonly used industrial material, sepiolite has been used as a therapeutic, functional, inert and bulk agent in the pharmaceutical and cosmetic industries (Carretero and Pozo 2010) and as an adsorbent to reduce the bitterness of cold-pressed grapefruit seed oil (Guneser and Yilmaz 2017). It has also been applied in the sorption of PTEs to improve environmental conditions (Álvarez-Ayuso and García-Sánchez 2003; Kocaoba 2009; Lazarević et al. 2007).

Sepiolite [Mg_8_Si_12_O_30_(OH)_4_·8H_2_O] is a complex hydrated magnesium silicate with a porous fibrous structure composed of two blocks of tetrahedral silica sheets and a sandwich octahedral sheet of magnesium hydroxide or oxide (Xu et al. 2017). Sepiolite is widespread and abundant in the earth and most research focuses on the application of natural sepiolite or purified products to remove pollutants from the environment. In addition, applications of purified, or modified, sepiolite are found in improving mechanical properties of materials, improvement of environmental quality (e.g. air) and in craftwork. There is also an increased interest in modified sepiolite materials for environmental treatment, exploiting higher performance characteristics that can be obtained (Padilla-Ortega et al. 2013).

The extraction and purification of sepiolite require energy input and with the current focus on the climate emergency, the process alone does not meet full circular economy objectives. To address these, the potential of resolving pollution problems and maintaining a low carbon footprint, a study is required that considers the whole life cycle including maximizing the residual value of material classified as waste materials from the extraction and the purification of sepiolite to provide more environmentally friendly and energy-saving methods for contamination treatment. We have previously considered the context of sepiolite as a potential technology to treat localized contamination in China (Wang et al. 2018), highlighting capacity and treatment opportunities for PTEs in aquatic systems, modification and regeneration issues. This review provides a perspective on the life cycle of technology considering the potential application of the raw material, exploitation of residues, properties, and possible risk with the application of sepiolite in projects for the treatment of contaminated soil or land.

## Geographical distribution of sepiolite

Suitability of structural features and properties of sepiolite make them very versatile materials that can be applied in many industries and thereby essential and crucial for the world economy as a whole. Spain is the world’s largest producer of sepiolite. In Europe, 90% of sepiolite comes from Spain, and the rest of the sepiolite comes mainly from Turkey. Tblsa SA is the largest producer of sepiolite in Spain with a production capacity of 700,000 tons/year, providing 515,000 tons of sepiolite products: of which about 353,000 tons are used in cat and pet litters, and about 162,000 tons are consumed as industrial adsorbents, carriers for chemicals (e.g. pesticides), animal feedstuffs (Yi 2003). The price of sepiolite products depends on particle size, degree of modification, taste, additives, treatment methods. Sepiolite prices vary depending on the degree of processing, as a guide, typical examples from the Spanish markets give low-grade processed sepiolite products ~ $32/t, industrial products ~ $112/t, rheology modifier products ~ $934/t, and specialized modified sepiolite products ~ $1871/t (Yi 2003). Raw sepiolite prices from China and Turkey are $180–300/t and $500–800/t, respectively, on Alibaba website: (https://www.alibaba.com/trade/search?fsb=y&IndexArea=product_en&CatId=&SearchText=raw+sepiolite&SearchScene=exhibition&spm=a27aq.14827689).

China hosts one of the world’s major sepiolite reserves (Yin et al. 2011). The wide applicability and utility of sepiolite in the industry for rheological and catalytic purposes is well known and has driven effort for industrial exploitation for over 200 years (Martin Vivaldi and Robertson 1971). There is a potential in sepiolite-based coating material that helps with arresting heat transmission to reduce energy consumption in daily lives (Wang et al. 2009). Deposits of Hunan province comprise weathered Marine-sedimentary formations, consisting of sepiolite-palygorskite beds in the upper layer of the Permian Qixia (Chihsia) formation and have provided raw materials for purification of high-quality sepiolite products for industrial application. Information on the global distribution of sepiolite deposits is given in Table 1. The chemical composition of the range of sepiolite sources is given in Table 2.

**Table 1:** Global sepiolite distribution

	Distribution	Country	Deposit	Deposition type	Estimated reserve (ton)	Approximately annual output (ton)	Study
1	North America	USA	Amargosa, Nevada	–	–	10000 (in Georgia)	(Eberl et al. 1982; Khoury et al. 1982; Liang 2008)
2	Europe	Spain	Vallecas-Vicάlvaro-Yunclillos, Batallones and Mara	–	–	700,000	(Mayayo et al. 1998; Yi 2003)
3	Western Asia	Turkey	Eskisehir	Marine sepiolite-palygorskite	17,000 (nodular sepiolite) for Eskisehir	20,000–60,000 (in 1990s) for Turkey	(DPT 2001; Hüseyin and Bozkaya 2011; Yalçin and Bozkaya 1995)
				Lacustrine sepiolite-palygorskite			(Yalçin and Bozkaya 1995)
				Hydrothermal sepiolite			(İrkec and Ünlü 1993)
				Hydrothermal sepiolite			(Yalçin and Bozkaya 2011)
			Anatolia	–	8.5 million for central Anatolia		(Hüseyin and Bozkaya 2011)
4	East Asia	China	Hunan, Henan	–	20 million for Hunan	–	(Wang et al. 2018; Zhang and Yang 1986)
5	East Africa	Kenya-Tanzania	Amboseli	–	–	320	(Liang 2008; Stoessell and Hay 1978)
6	Africa	Somalia	EI-Bur	–	–	Hundreds	(Liang 2008; Singer et al. 1998)
7	The whole world				–	850,000	(Galán and Pozo 2015)

**Table 2:** Sepiolite composition across the world

	SiO_2_	TiO_2_	Al_2_O_3_	Fe_2_O_3_	FeO	MnO	CaO	MgO	K_2_O	Na_2_O	H_2_O^+^	H_2_O^−^	LOI	Total	Study
1	59.41	0.02	0.42	0.76	0.02	0.08	1.47	24.03	0.17	0.05	9.22	9.68		105.33	(Zhang and Yang 1986)
2	60.88	0.002	0.45	0.48	0.12	0.004	0.08	25.68	0	0.03	11.68	9.5*		99.41	(IGGCMBMI 1980)
3	58.71	0.02	0.9	1.07	0.09	0.06	1.86	25.06	0.1	0.35	9.75			99.43	(Zhang and Yang 1986)
4	61.4	–	0.38	0.19	0.07	–	0.79	26.51	< 0.01	0.03	11.17	9.45*		100.55	(IGGCMBMI 1980)
5	52.69	0.03	0.41	0.44	0.2	0.1	7.2	24.29	–	–	8.35		14.51	99.8	(Zhang and Yang 1986)
6	55.10	0.05	3.13	0.04	0.01	0.04	0.23	22.37	0.16	0.08	7.40	11.40		100.01	(Peng 2017)
7	54.44	–	0.4	0.15	0	–	0.11	24.59	0	0.05	9.12	11.5		100.39	(Zhang and Yang 1986)
8	54.75	–	0.8	4.1	–	–	1.68	18.3			20				(Liu and Xu 1984)
9	55.21	0.05	0.43	0.15		0.02	0.2	24.26	0.15	0.1	–	–	19.21	99.78	(Lopez Galindo et al. 1996)
10	63.10	–	1.08	0.27	–	–	0.49	23.80	0.21	0.09	10.88	–	–	99.92	(Galan and Castillo 1984)
11	53.17	0.17	1.76	0.99	0.04	–	0.23	24.70	0.97	0.45	8.29	8.79	–	99.57	(Hay and Stoessell 1984)
12	56.46	0.08	1.17	0.37	–	–	0.19	22.67	0.18	0.06	–	–	18.57		(Pozo et al. 2014)
13	56.95	0.15	1.05	0.93	–	–	2.45	23.35	0.41	0.11	–	–	14.60		(Ece and Çoban 1994)

*The total amount does not include H_2_O^−^

Fibers sepiolite ($\alpha$-type): (1) Henan Neixiang; (2) Henan Lushi; (3) Anhui Quanjiao; (4) Hubei Guangji; (5) Zhejiang Lin’an; (6) Hunan Yonghe; (7) Sichuan Shimian; (8) Guangxi Du’an; (13) Turkey Eskişehir

Clay sepiolite ($\beta$-type): (9) Spain Madrid; (10) Spain Vallecas; (11) Kenia Amboseli; (12) Spain Batallones; (13) Turkey Eskişehir

## Sepiolite-based material single application

### Polymer nanocomposite sepiolite

#### Application in machinery

Sepiolite has remarkable adsorptive properties as a result of high surface area, and therefore it has been widely used in the preparation of polymer nanocomposites preparation as it increases dimensional stability, processability, thermal resistance and mechanical strength. The influence of different nanofillers, such as sepiolite, has been studied on the properties and characteristics of natural rubber nanocomposites, which showed the maximum improvement in modulus because of the high surface energy of sepiolite which is 38 mJ/m^2^ (Bhattacharya et al. 2009).

The effect of sepiolite nanoclay on the tack strength of brominated isobutylene-*co*-*p*-methyl styrene loaded rubber was studied (Kumar et al. 2010). It was revealed that tack strength increased by around 300% over that of neat BIMS (brominated isobutylene-*co*-*p*-methyl styrene) rubber. The molecular diffusion across the interface of BIMS rubber was lowered upon the addition of sepiolite nanoclay due to its reinforcing effect. However, the average penetration depth of the diffused chains of BIMS in the composites was still sufficient to form entanglements at either side of the interfaces, leading to greater resistance to separation due to an increase in cohesive strength of the corresponding nanocomposites (Kumar et al. 2010).

Sepiolite was modified by Tartaglione et al. (2008) in two different processes of functionalization: surface adsorption of quaternary ammonium salts/amines or grafting of silane reagents to surface silanols by covalent bonding. The grafted sepiolite-based nanocomposites of poly (butylene terephthalate) (PBT) and polypropylene (PP) are performed higher thermal stability compared to those of adsorbed sepiolite. There is no effect of sepiolite on thermal characters of PBT, whereas there is a polymer crystalline structure revealed on PP nanocomposites. Sepiolite was modified with cetyltrimethyl ammonium bromide (CTAB) by ion exchange and further was used to prepare polypropylene and polypropylene grafted with maleic anhydride composites by melting extrusion. The properties of modified sepiolite were compared with those of unmodified sepiolite-based nanocomposites. It is sepiolite that was modified by polypropylene/polypropylene grafted maleic anhydride/CTAB, showing the best interaction and resulting in increased elastic modulus and thermal resistance. There is a lower maximum heat release rate for the composite than those of neat polymer.

#### Application in wastewater

In wastewater treatment, sepiolite has been applied as a denser core anchoring chain for a polyelectrolyte, such as a polycationic polymer, with charge opposed to the colloidal charges, which leads to the fast formation of neutralized flocs with a higher density than organic colloids (Rytwo 2014, 2017). Sepiolite polymer nanocomposites offer effective clarification of water with a reduction of turbidity and suspended solids by more than 90% in effluents (Rytwo 2014, 2017). It is a technology that could be developed for wide ranges of applications. The properties of the polymer will be combined with that of the fillers, which means the significance of modification is obvious.

A novel amidoxime based chelating nanohybrid adsorbent was synthesized with poly (acrylonitrile) grafted sepiolite nanohybrid material (RGS) with simultaneous radiation grafting technique (Taimur et al. 2017). Batch adsorption studies were carried out for Cu adsorbed on amidoximated nanohybrid adsorbents to study the effects of pH, contact time, adsorbent dose and initial concentration. Removal of Cu ion attained equilibrium follows pseudo-second-order kinetics within 30 min. There is a good description of the equilibrium process by the Langmuir isotherm model, and adsorption capacity reached its maximum at 278 mg/g for irradiated samples (Taimur et al. 2017).

Polyaniline/sepiolite (PANI/sepiolite) nanofibers were synthesized by chemical oxidation polymerization to remove Cr^6+^ from aqueous solution (Chen et al. 2014). The adsorption of Cr^6+^ to the PANI/sepiolite nanofibers was strongly influenced by pH within a range of 2–10, and adsorption efficiency of Cr^6+^ to the PANI/sepiolite reached the highest at pH 2. The maximum adsorption capacity of the nanofibers for Cr^6+^ was utmost at 206.6 mg/g at 25 °C, and it increased with the increase in temperature. Experiments of desorption revealed that PANI/sepiolite can be reused and regenerated for 2 continuous cycles without loss of its removal efficiency (100% in the first cycle and removal efficiency remained almost the same in the second cycle), which indicated that the nanofibers can be used as an economical and efficient adsorbent for Cr^6+^ (Chen et al. 2014).

Modified sepiolite combines with other fillers has been a subject of research and the work has been started. The opportunities for sepiolite applications are vast and hence there are numerous opportunities to utilize sepiolite. Along with its specific characteristics, both the long-term durability and the cost-effectiveness of the application need to be considered, making it possible to design ready-to-use products with good market acceptance. Sepiolite polymer nanocomposite has not been applied in the treatment of polluted soil or land so far because it is difficult and expensive to retrieve the polymer nanocomposite for reprocessing/reuse.

### Other modified sepiolite materials for treatment

#### Application in water

To improve PTEs adsorption to sepiolite, there are a number of modification methods, including acid treatment, thermal treatment, magnetic modification, organic-modification and acid-thermal treatment (Wang et al. 2018). A method for removal of PTEs using nanoscale-zero-valent iron material loaded with sepiolite in aquaculture water involves microwave-assisted acid modification of sepiolite powder in hydrochloric acid solution. Therefore, it can quickly adsorb and remove PTEs from the aquaculture water body, help to adjust the pH to meet the requirements for good aquaculture conditions. The reaction conditions required are relatively easily achieved, the raw materials are low cost and easy to obtain, and this method is overall less time-consuming compared with traditional chemical precipitation of PTEs. The reaction is mild and the method is suitable for wider operation (Liu et al. 2018). In addition, to treat Cu in aquaculture wastewater, negatively magnetically modified sepiolite has been used to adsorb the Cu ions followed by filtering the sepiolite adsorbent and post-treatment processing (Zhang et al. 2018). A comparison of the adsorption capacity of waste sepiolite, modified sepiolite, natural sepiolite and other materials is given in Table 3.

**Table 3:** The adsorption capacity of sepiolite, modified sepiolite and other materials

Material	Treatment	PTE (s)	Capacity	Reference
Waste sepiolite	–	Boron	96.15 mg/g	(Öztürk and Kavak 2004)
Activated waste sepiolite	HCl activated	Boron	178.57 mg/g	(Öztürk and Kavak 2004)
Natural sepiolite		U	34.61 mg/g	(Donat 2009)
Modified sepiolite	Modified by 3-(trimethoxysilyl) propyl methacrylate (monolayer maximum adsorption capacity)	Cu, Mn, Zn, Fe, Co, Cd	12.3 × 10^−5^ mol/L, 11.7 × 10^−5^ mol/L, 9.0 × 10^−5^ mol/L, 8.2 × 10^−5^ mol/L, 5.7 × 10^−5^ mol/L, 1.8 × 10^−5^ mol/L	(Beyli et al. 2016a)
Modified sepiolite	Modified by N-1-[3-(trimethoxysilylpropyl)]	Co, Cd, Mn, Fe, Cu, Zn	30 mol/g, 37 mol/g, 80 mol/g, 110 mol/g, 118 mol/g, 118 mol/g	(Beyli et al. 2016b)
Activated carbon		Cd, Cr, Cu, Pb	0.19 mg/g, 0.16 mg/g, 0.21 mg/g 0.18 mg/g	(Sajjad et al. 2017)
Silver birch (*B. pendula*) biochar		Cu, Pb	0.13 mg/g, 1.29 × 10^−3^–3.77 × 10^−3^ mg/g	(Komkiene and Baltrenaite 2016)
Scots pine (*P. sylvestris L.)* biochar		Zn, Pb	0.11 mg/g, 2.37 × 10^−3^–4.49 × 10^−3^ mg/g	(Komkiene and Baltrenaite 2016)
Chitosan		As	50.0 mg/g	(Brion-roby et al. 2018)
Rice straw		Cr	34.90 mg/g	(Lin et al. 2018)
Chinese loess		Ni	15.61 mg/g	(Wang et al. 2015)
Modified agricultural wastes		Cd	195.5 mg/g	(Al Othman et al. 2011)

Studies have been carried out on the structural effect of cationic surfactant to treatment capacity of PTEs (Kalpakli and Cansev 2018). The organo-clays were synthesized with smectite and hexadecyltrimethylammonium bromide (HDTMAB) which is a cationic surfactant. Moreover, the characteristics of prepared organoclay to remove Cd^2+^, Cu^2+^ and Pb^2+^ ions from solution have been studied and the results demonstrated that organoclay increased their adsorption capacity for the Pb, Cu and Cd from aqueous solution (Kalpakli and Cansev 2018). A sepiolite modified material was prepared by microwave reaction of the mixture of sepiolite and kaolin and the mixture of ferric salt of cellulose, ferrous chloride and ferric chloride and then applied to remove Cd^2+^, Cu^2+^, Cr^6+^ and Pb^2+^ ions from solution with the benefit of a lower cost, higher adsorption efficiency and stability of the material (Huang 2017).

Composites of natural sepiolite (SEP) and partially acid-activated (AAS) were prepared at different ratios and nanoscale zero-valent iron (nZVI) to obtain the best nZVI dispersibility and best performance of adsorption of Cd^2+^ (Habish et al. 2017). Though there is a higher surface area and pore volume of nZVI composed with AAS, the dispersibility of nZVI was better when SEP was used as the support. However, a lower degree of oxidation was attained during the synthesis by AAS. X-ray photoelectron spectroscopy (XPS) analysis of the composite with the best dispersibility for nZVI before and after adsorption of Cd^2+^, indicated that the surface of the nZVI particles was composed of oxidized iron species. The adsorption isotherms of Cd^2+^ revealed that the existence of SEP and AAS reduced the agglomeration of nZVI particles when being compared to the pure nZVI, which showed higher adsorption capacity (Habish et al. 2017). A study was carried out by Bahabadi et al. (2017) on the application of natural sepiolite and Fe^3+^ modified sepiolite for the treatment of Cu, Zn and Cd in aqueous solutions. The concentration of iron chloride applied for modification was 1 M. Zn removal by sepiolite remained the same before and after the surface modification, while removal of Cu and Cd were increased by 13.6% and 21.2%, respectively.

A modified sepiolite was prepared with magnetite by chemical co-precipitation, and the magnetite/sepiolite (M/SEP) composite was characterized by X-ray diffraction (XRD), scanning electron microscopy (SEM), Fourier transform infrared spectroscopy (FT-IR), vibrating sample magnetometer (VSM) and X-ray photoelectron spectroscopy (XPS). The results revealed the composition of M/SEP is magnetite and sepiolite, having a superparamagnetic property. The results of the application of M/SEP to adsorb Co^2+^ and Cd^2+^ from aqueous solutions showed that sorption processes were endothermic and spontaneous processes on the basis of thermodynamic parameters, which indicates that M/SEP composite can be used on the preconcentration of Co^2+^ and Cd^2+^ in wastewater treatment (Yu et al. 2016).

Furthermore, sepiolite was also modified by *N*-1-[3-(trimethoxysilylpropyl)] and the interaction with PTEs was studied systematically as a function of ionic strength, pH and temperature using ASS. The results revealed that the amount of adsorbed PTEs increased with an increase in equilibrium pH and temperature of the solution, while it decreased generally with the increase in ionic strength. The adsorption mechanism of metal ions to modified sepiolite was identified by analyzing the experimental data on the basis of Langmuir and Freundlich isotherms, which indicated that the isotherm data were correlated to Langmuir isotherm. The monolayer adsorption capacities of modified sepiolite were estimated as 30, 37, 80, 110, 118 and 118 mol/g for Co^2+^, Cd^2+^, Mn^2+^, Fe^3+^, Cu^2+^ and Zn^2+^, respectively. The affinity of adsorption was in the order of Co^2+^ < Cd^2+^ < Mn^2+^ < Fe^3+^ < Cu^2+^ = Zn^2+^. The results demonstrated that Co^2+^ and Cd^2+^ were less efficiently adsorbed than Fe^3+^, Cu^2+^ and Zn^2+^ by modified sepiolite. In conclusion, modified sepiolite can be applied as an alternative low-cost adsorbent in adsorption process in nanocomposite synthesis and chromatographic purification process (Beyli et al. 2016b).

Sepiolite was synthesized by nanoscale zero-valent iron particles (S-NZVI) for the adsorption and reduction of Cr^6+^ and Pb^2+^ ions from groundwater and results showed that S-NZVI had the potential for in situ remediation of heavy metal-contaminated groundwater (Fu et al. 2015). Acid-activated sepiolites and natural sepiolites were functionalized with covalent grafting [3-(2-aminoethylamino) propyl] trimethoxy-silane on the sepiolite surface, and their adsorption capacities of amine-functionalized sepiolites to Cr^6+^ from aqueous solution were investigated (Marjanović et al. 2013). The adsorption isotherms showed that the adsorption capacities of the modified acid activated sepiolite were higher than those of modified natural sepiolite for all of the researched initial pH values. The maximum concentrations of Cr^6+^ removal were 60 mg/g of functionalized acid-activated sepiolites and 37 mg/g of functionalized sepiolites, respectively, achieved at an initial pH of 2.0 (Marjanović et al. 2013).

#### Application in soil

A mercapto functionalized sepiolite material (MSEP) was prepared as an immobilization agent for treatment of Cd-polluted paddy soil (Liang et al. 2017). The Cd content of husked rice (*Oryza sativa* L.) was reduced by 65.4–77.9% at the trace dosages of 0.1–0.3% for MSEP. Single and sequential procedures showed that MSEP could boost the sorption or fixation of Cd on soil and decrease the bioavailability of Cd (Liang et al. 2017). MSEP had negligible effects on the point of zero charge of the paddy soil and pH values. There is no obvious effect on extractable Zn, available phosphorus and hydrolysable nitrogen in the soil, which indicated that MSEP is environment friendly and compatible to use (Liang et al. 2017).

All of the sepiolite-based material might have a good effect on soil remediation; however, reuse or recycle of products is a problem. Furthermore, it might be a potential threat that some chemicals of the products could enter into groundwater.

## Sepiolite combined with other additives in treatment

Raw sepiolite, modified sepiolite and sepiolite tailings have been applied as an additive combined with other additives together, which is adsorbent for water treatment, amendment agent, conditioner agent and inactivator for soil remediation. For instance, for PTEs polluted soil application of sepiolite-based material has included adding 10–385 wt% modified sepiolite tailings, 1–80 wt% modified zeolite, 1–5 wt% hyposulfite, 10–20 wt% nano iron powder and 45–60 wt% quicklime (Wang et al. 2017).

**Fig. 1:**
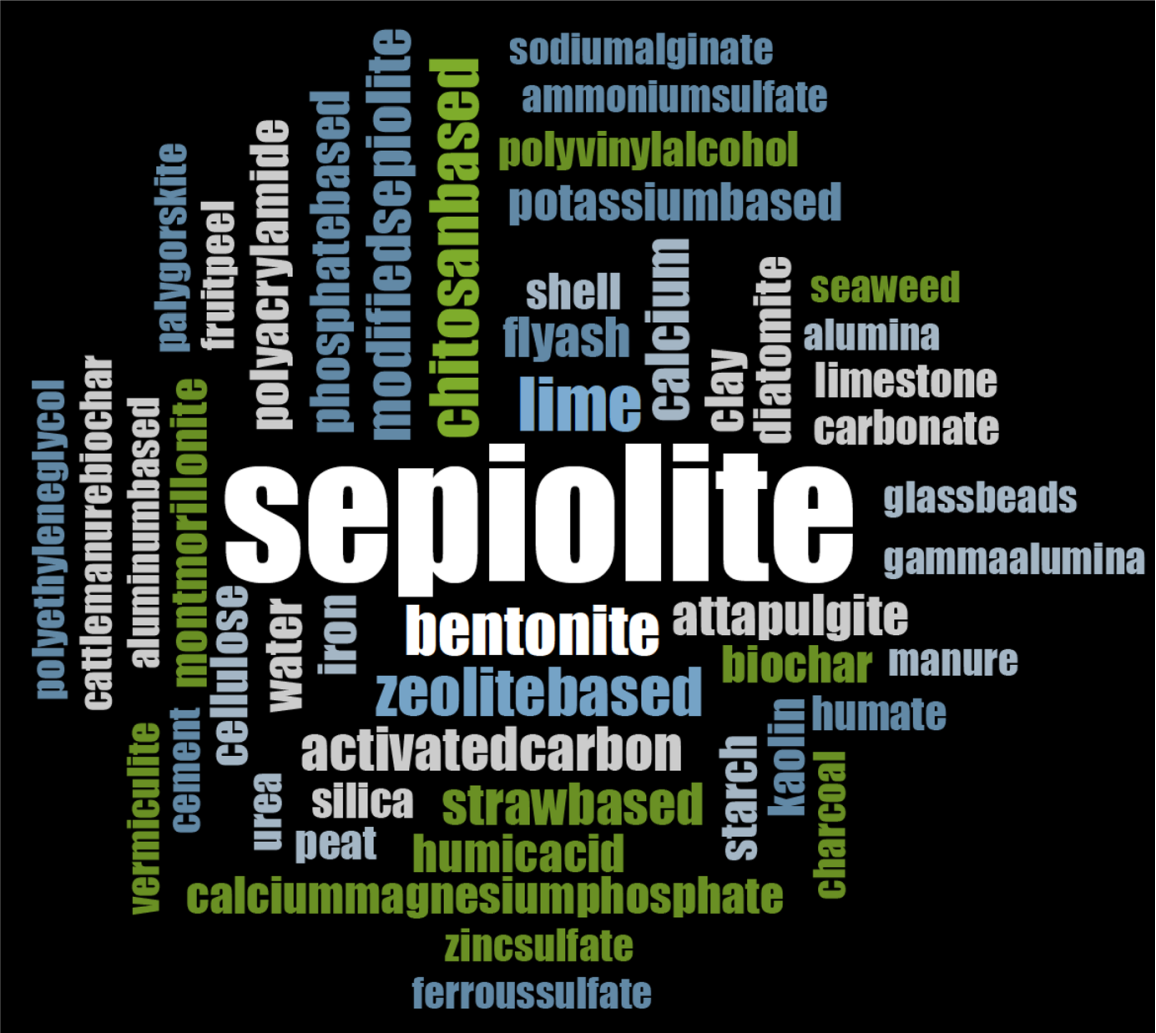
A picture of word cloud to show materials appeared in proportion for PTEs treatment

**Fig. 2:**
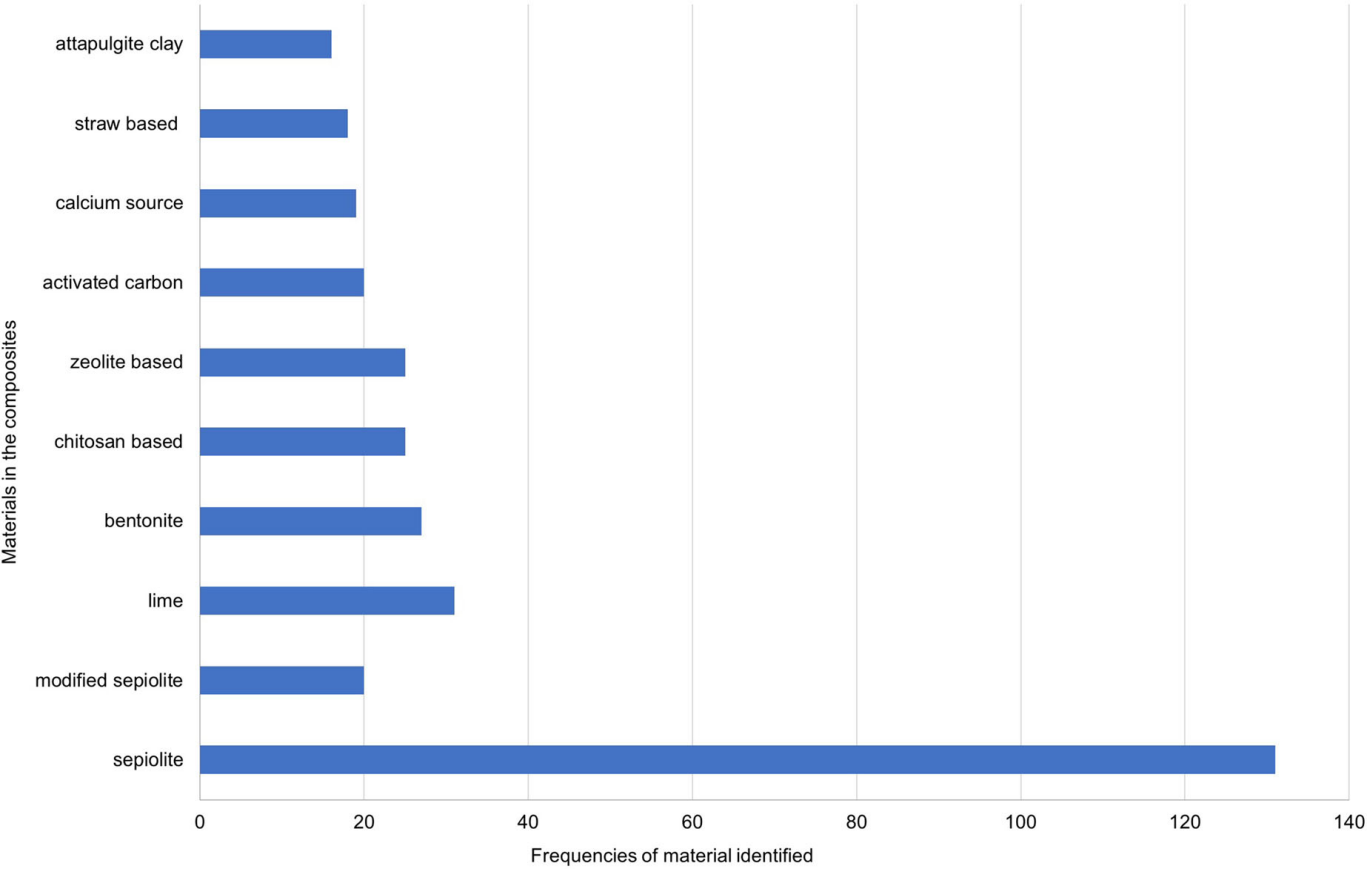
Variation of additives in sepiolite-based composites (in literature reports 1864–2018)

**Fig. 3:**
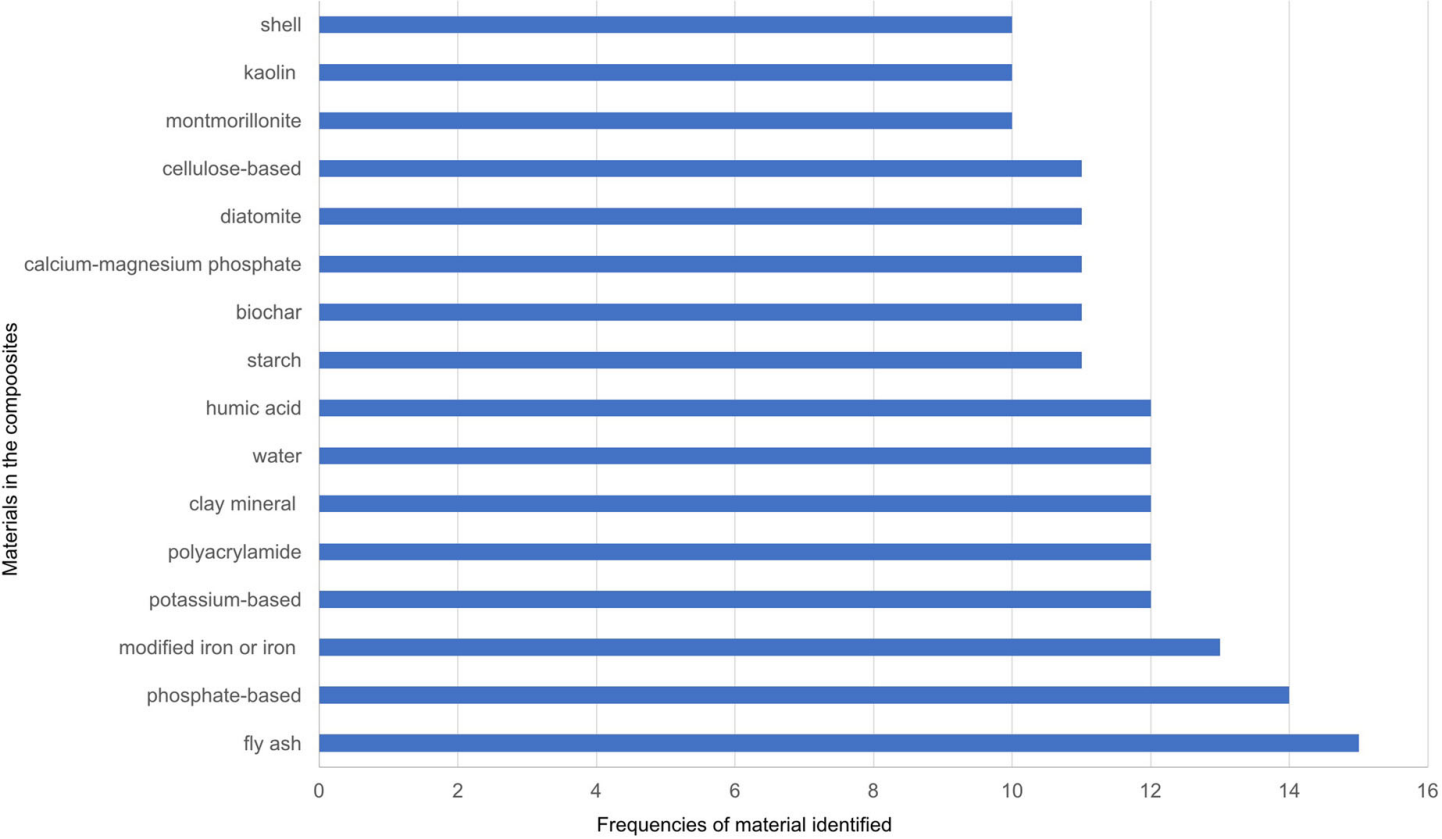
Variation of additives in sepiolite-based composites (in literature reports 1864–2018)

**Fig. 4:**
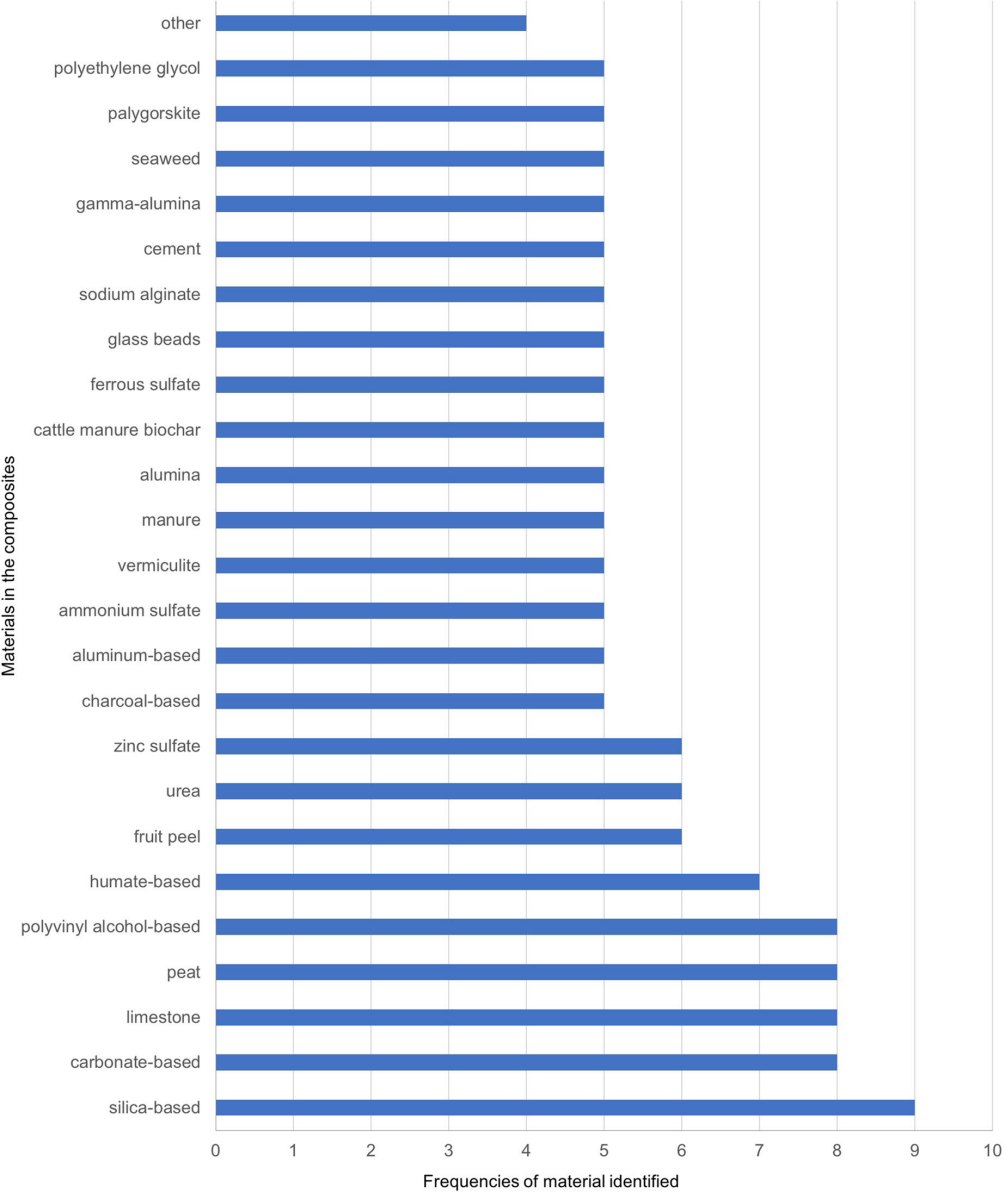
Variation of additives in sepiolite-based composites (in literature reports 1864–2018)

We identified 150 sepiolite-based literature reports and patents from databases (Web of Science, United States Patent and Trademark Office (https://www.uspto.gov), Chinese Patent database (http://www.soopat.com/) for application in the treatment of water or soil contaminated by PTEs. The word cloud picture (Fig. 1) is produced by software Nvivo12 with an analysis of key content of combinations of materials. Figures 2, 3 and 4 are produced from Microsoft Office 2010 Excel by frequencies analysis to all additives of combinations of materials. All of them get checked if they (natural sepiolite, modified sepiolite, sepiolite tailings) are used as an additive to combine other additives for PTEs treatment in water, soil, land, sludge. There are published papers and patents (English abstract from different countries) in the database Web of Science. Only published patents and licensed patents are shown in the US patent office (in English) and Chinese patent database (in Chinese). A patent is only counted as one though this one might be published and licensed in more than one document.

To synthesize a large amount of literature and patents published on sepiolite, a word cloud picture has been prepared (Fig. 1). Figures 2, 3 and 4 describe the results in more detail.

A total of 10 materials appear with the frequency of application between 16 and 140 times used in the formation of composites with sepiolite, followed by lime (31), bentonite (27), chitosan based (25), zeolite based (25), activated carbon (20), modified sepiolite (19), calcium source (19) and straw based (18) (Fig. 2). We identified 16 materials with a frequency of application between 10 and 16, the highest being fly ash (15), followed by phosphate-based (14), modified iron or iron (13), while potassium-based, polyacrylamide, clay mineral, water and humic acid showed the same frequency (12), followed by starch, biochar, calcium–magnesium phosphate, diatomite and cellulose-based (11), montmorillonite, kaolin and shell (10) (Fig. 3). There are 24 materials in the frequencies of 5–9, with the highest frequency is silica-based (9), followed by humate-based (7), while carbonate-based, limestone, peat and polyvinyl alcohol-based were at the same frequency (8), followed by fruit peel, urea and zinc sulfate (6), charcoal-based, aluminum-based, ammonium sulfate, vermiculite, manure, alumina, cattle manure biochar, ferrous sulfate, glass beads, sodium alginate, cement, gamma-alumina, seaweed, palygorskite and polyethylene glycol (5). In addition, the frequencies of 1–4 from all other kinds of materials are included in the other figure (Fig. 4).

Fly ash or coal fly ash is an industrial by-product generated from the combustion of coal for thermal power generation and other combustion activities (Ahmaruzzaman 2010). In addition, bottom ash and lagooned ash are formed during the combustion of coal. Bottom ash shows a similar phase, particle morphology and chemical composition with fly ash though the unburnt coal component and proportion of amorphous particle is greater than that of fly ash. Lagooned ash shows an intermediate, particle morphology and phase between fly ash and bottom ash (Vassilev 1992). There are mainly two classes of fly ash, class F contains more than 8% CaO and class C contains less than 8% CaO. Both contain various oxides Al_2_O_3_, SiO_2_, Fe_2_O_3_, Na_2_O, MgO, MnO, P_2_O_5_ and TiO_2_. Other elements (Cu, Pb, Cd, Cr) and radioactive isotopes (^232^Th, ^238^U, ^40^K) can be also found in some fly ash (Wang et al. 2020).

Emissions and accumulation of fly ash not only occupy land, but also cause serious pollution to the environment and poses a disposal challenge (Ochedi et al. 2020). However, fly ash is also a resource that can be widely applied in the fields of construction materials, road sub-base, mine backfill etc. (Ahmaruzzaman 2010), and fly ash also shows a promising ability as a heterogeneous catalyst to oxidize aqueous Na_2_S solution (Mallik and Chaudhuri 1999) or sorbent to remove various air pollution (Ahmaruzzaman and Gupta 2012; Ochedi et al. 2020). Fly ash is utilized as a constituent of mixtures for solidification/stabilization technologies because of its low-cost, good pozzolanic properties, which helps with solid waste disposal and coping with potential stress of its large generation and accumulation in an economically beneficial way (Terzano et al. 2005).

The influence of fly ash on properties of sepiolite is carried out by adding fly ash to the sepiolite with 2%, 4% and 6% by weight (Joseph and Angel 2020). The results showed that the pH of the mixture is decreased with a content increase in fly ash. The specific gravity and the Atterberg limit of the mixture showed a negative trend with an additional increase in fly ash. The mechanical standard compaction test results revealed a slight increase in the maximum dry density and a decrease in the optimum moisture content with an increase percentage of fly ash in the mixture. The strength and permeability of the mixture were improved up to 4% of the fly ash content, after that they showed a decrease in trend. The maximum values of strength and permeability of the sepiolite were reached at 4% addition of fly ash (Joseph and Angel 2020).

All of the sepiolite-based material additives might have a good effect on water treatment which is recyclable and low cost. They are much safer and cheaper to be added in soil but could still be more difficult to control the amount of sepiolite-based material and combined ratio because of various soil background comparing with other soil remediation methods.

## Economic constraints for the application of sepiolite in remediation

### Direct project cost and period of treatment

In the research of potential for ensuring the safety of production of rice planted in Cd-polluted soil by using sepiolite and humic acid, the production was studied using *Oryza sativa* L. (Jia-33) in which the accumulation of Cd with different weights of additives (including humic acid at 0.750, 2.250, 3.750, 5.250, 7.500 t/ha; or sepiolite at 2.250, 6.750, 11.250, 15.750, 22.500 t/ha, or combination of humic acid and sepiolite at 0.375 + 1.125, 1.125 + 3.375,1.875 + 5.625,2.625 + 7.875, 3.750 + 11.250 t/ha) was assessed (Xie et al. 2018). Results showed that applications of humic acid are at 5.250 t/ha, or sepiolite at 6.750 t/ha or a combination of humic acid at 1.125 t/ha and sepiolite at 3.375 t/ha leads to that Cd content of polished rice were 0.171 ± 0.01 mg/kg, 0.184 ± 0.01 mg/kg, and 0.181 ± 0.01 mg/kg, respectively, lowering than the Cd limit defined in the National Food Safety Standard (GB 2762-2012, 0.2 mg/kg) of the PRC. The material purchase prices were 197.59 $/t of humic acid, 112.91 $/t of sepiolite, which means that the corresponding cost of applications of 5.250 t/ha humic acid, 6.750 t/ha sepiolite, the combination of 1.125 t/ha humic acid and 3.375 t/ha sepiolite were 1037.34, 762.13 and 433.99 $/ha, respectively (Xie et al. 2018).

Research has shown that Cd passivated by the soil amendment calcium–magnesium phosphate and lime starts to re-release from the fourth year, which revealed that the passivation effect could last for 3 years (Jiang et al. 2017). This is much better than more intrusive stabilization methods such as lime/cement as soil function is preserved to some extent. Regardless of human resource and in terms of the cost of the materials, the lowest cost for the combination of humic acid at 1.125 t/ha and sepiolite at 3.375 t/ha with the average annual cost is about 144.66 $/ha. This further illustrates the potential of low accumulation strains of rice combined with the application of the sepiolite to meet agricultural safety standards (Xie et al. 2018).

### Remediation potential for contaminated land

Sepiolite is applied as amendments or immobilizers for remediation of soils contaminated with PTEs. The remediation efficiency and function of sepiolite on the remediation Pb and Cd-polluted farmland have been verified with field studies by Zhan et al. (2019). The pH value of soils increased by 1.67 after the application of sepiolite, whereas the CaCl_2_-extractable concentration of Cd and the exchangeable concentration of Pb in soils decreased by 63.4% and 21.5%, respectively. The results indicated that immobilization mechanisms for the sepiolite may be complex, which not only involved with surface adsorption due to high specific surface but also involved with a high ion-exchange capacity of sepiolite (Zhan et al. 2019).

Sepiolite materials alone or in a combination with other materials such as limestone/bentonite/calcium carbonate can significantly cut down the content of Cd in brown rice, regardless of its application in a field study or pot trials (Sun et al. 2016a, b; Wu et al. 2016; Li et al. 2020). For instance, when sepiolite combined with low Cd-accumulating cultivar rice (*Oryza sativa* L.) in field experiment, the concentration of Cd in brown rice of low Cd-accumulating cultivar rice (*Oryza sativa* L.) from the control group was 0.72 mg·kg^−1^, while the concentration of Cd in brown rice of the cultivar rice from the experimental group was 0.18 mg/kg, which is lower than that of the maximum amount lever proposed as “Maximum Levers of Pollutants in Foods” standard (GB 2762-2012) in the Chinese national standard and the Codex Alimentarius Commission (CAC 153-1995) requirement in the World Health Organization (WHO) (Liang et al. 2014).

However, there are differences in remediation effects of sepiolite-based amendment on soil polluted with Cd, Pb. For example, a 3-year in situ experiment was carried out in a paddy soil around a mine of southern Hunan, China (Wu et al. 2016). The combined amendment (LS, limestone and sepiolite) was applied in order of 0, 2, 4 and 8 g/kg (w/w) and rice were subsequently planted in 2012 (first season), 2013 (second season) and 2014 (third season). The results showed that the pH values of soils increased significantly in all three seasons and the ranking of enhancement was as follows: the first season > the second season > the third season. Experiment results indicated that the effect of the LS on reducing exchangeable Pb and Cd content of soil ranked as follows: the first season (97.6–99.8%) > the second season (80.7–97.7%) > the third season (32.6–97.7%) for Pb and the first season (88.3–98.9%) > the second season (28.3–88.0%) > the third season (8.3–71.4%) for Cd, respectively. It showed that the effects were persistent on soil exchangeable Cd concentration but gradually decreased with time on soil exchangeable Pb concentration, which implied that LS was more suitable for Cd than Pb contaminated soil in long-term remediation (Wu et al. 2016). Furthermore, a 2-year consecutive experiment was conducted on immobilization effect of sepiolite alone by Liang et al. (2016), which revealed that immobilization effect is maintained seen in the decrease in Cd content of the rice, 0.025 M HCl extractable Cd content of paddy soil and the soil exchangeable Cd in the second year without any additional amendment.

### Economic impacts and project risk

In China, contaminated land is identified by the government or local authority. Typically, several contamination treatment enterprises will compete via tender to secure a pollution treatment project. Once a firm is appointed, a feasibility report for soil remediation is required for the company to obtain final approval from the relevant authority. The specific details of the project are then decided; this involves a comparison of remediation technologies and methods. The project would be carried out after the approval is granted; then immediate or long-term monitoring of contamination treatment results would be arranged based on the design.

To use sepiolite for the treatment of land contamination by PTEs will increase the value or quality of the land and its potential possibility for reuse commercial purpose, create more space for residential development in the area, increasing employment in land remediation and redevelopment, which bring more local tax revenues (Song et al. 2019). With the development of society and new knowledge gained by individuals, the choice of methodology for soil remediation could be combined with positive interactions with neighborhoods, for example through community center development as part of the remediation, which can satisfy the social and economic needs of different stakeholders. This would support the harmonious integration of new solutions for contamination treatment within surrounding communities and lead to a more sustainable local economy (Dagenhart et al. 2006).

When remediation projects involved some related risk, the regular lifestyle might be impacted, affecting local traffic movement, environmental degradation during project implementation, or for workers’ safety during the remediation period and even neighbor nuisance complaints. Recent studies of a farmland remediation project (Xu et al. 2019) showed that by adopting species sensitivity distribution models with joint probability curves, a different conclusion was reached related to ecological risk assessment results compared to that of the traditional methods. The conclusion revealed that Cr indicated the highest risk at 84.3% that presents an 84.3% probability for 5% of the species to exceed NOEC/LOECs (maximum no-effect concentration and the lowest effective concentration) in Zhuzhou City (Xu et al. 2019). This study showed that the soil ecological risk of exposure to PTEs is underestimated by traditional methods and prioritizing ecosystem protection could be considered for PTEs contaminated land treatment (Xu et al. 2019). However, in the site where sepiolite-based material is applicated, microbial community diversity and soil enzymatic activities showed that soil metabolic function is a certain degree recovered and sepiolite application is environmentally friendly (Sun et al. 2016b).

Ultimately sepiolite, its modified forms and components of the recovered waste provides remediation capability by immobilizing contaminants and restricting migration in the aqueous phase. This reduces food chain transfer and may be enhanced by combining with various additives. However, when considering the complex characteristics of the soil, the effectiveness and durability of sepiolite-based materials could be decreased with low pH (Chen et al. 2020), releasing pollution and making PTEs bio-accessible to crops and even increase the risk to humans. Constant changes in soil environment and soil quality will continue to present new challenges to application amount control of products and monitoring by environmental laboratories. The use of sepiolite as an additive for soil remediation may require additional testing that has not included within regulatory methods.

## Summary, conclusion and future perspectives

This review highlighted the geographical distribution of sepiolite, its application for PTEs treatment in soil and land, assessing approaches to modification and composition on the application of sepiolite-based systems within site remediation and wastewater treatment. The considerable potential is highlighted for modified sepiolite or natural sepiolite to be applied in combination with other treatment methods. These should not be viewed in isolation, and an integrated assessment is needed to identify the future influence on the economic feasibility and if true environmental control is obtained.

A life cycle approach to sepiolite-based technologies needs to be developed. Commencing with natural sepiolite mining and exploitation, the pretreatment, purification and modification coupled with the waste generated at each stage. Modified and activated sepiolites are products that are utilized as soil conditioners, soil amendments or pesticide carriers that reside in the soil and become integrated part of the soil. Waste materials from mining exploitation and purification could be potentially used alone or combined with other additives in the treatment of locally slightly contaminated soil or to be soil fertilizers.

Sepiolite applications can have economic advantages relative to other contamination treatment methods, such as smaller project costs, more environmentally friendly impact on contaminated land, and reduced project risks. The treatment period of sepiolite applications also compares favorably with other treatment methods, such as phytoremediation and constructed wetlands. Sepiolite application combined with other low PTEs accumulating plants has the potential for cost effective solutions. However, more investigation is needed to clarify the appropriate scale of land application, development of technology to meet that and the potential for regulation change to allow stabilization and immobilization of the environmental hazard.
